# Spatial Analysis of Skilled Birth Attendant Utilization in Ghana

**DOI:** 10.5539/gjhs.v6n4p117

**Published:** 2014-04-13

**Authors:** Benedict O. Asamoah, Anette Agardh, Ellen K. Cromley

**Affiliations:** 1 Social Medicine and Global Health Division, Department of Clinical Sciences, Lund University, CRC, Malmö, Sweden; 2 Department of Occupational and Environmental Medicine, Laboratory Medicine, Lund University, Skåne University Hospital, Lund, Sweden

**Keywords:** maternal health, skilled birth attendant, utilization, spatial analysis, geographical patterns, Ghana

## Abstract

**Background:**

Maternal mortality is a major health problem in most resource-poor settings, especially in sub-Saharan Africa. In Ghana, maternal mortality remains high and births attended by skilled health professionals are still low despite the introduction, in 2005, of free maternal health care for all women seeking care in public health facilities.

**Objectives:**

This study aimed to explore geographical patterns in the risk of not utilizing a skilled birth attendant during childbirth in women of different socioeconomic backgrounds in Ghana.

**Methods:**

Global and Geographically Weighted Odds Ratios (GWORs) were used to examine the spatially varying relationships between low socioeconomic status (low education and low income) and non-utilization of skilled birth attendants based on data from the Ghana Demographic and Health Survey (GDHS) 2008.

**Results:**

Low education and low income were associated with non-use of skilled birth attendants. The GWORs revealed a north-south spatial variation in the magnitude of the association between non-use of skilled birth attendants and low education (Log GWOR ranged from 0.75 to 9.26) or low income (Log GWOR ranged from 1.11 to 6.34) with higher values in the north.

**Conclusion:**

The relationship between low socioeconomic status and the non-use of skilled birth attendants in Ghana is geographically variable. Effective governmental and non-governmental interventions are needed to address these regional inequalities.

## 1. Introduction

Maternal mortality remains a major health problem in most resource-poor settings, especially in sub-Saharan Africa (Hogan et al., 2010; Lozano et al., 2011). The United Nations Millennium Development Goal 5 (MDG 5) focuses on improving maternal health (UN, 2013). MDG 5a targets reducing the maternal mortality ratio by three quarters between 1990 and 2015.

The proportion of births attended by skilled health personnel (skilled birth attendants) is one of the main indicators used to monitor progress in reaching MDG 5. The World Health Organization (WHO), International Confederation of Midwifes (ICM) and International Federation of Gynecology and Obstetrics (FIGO) have defined a skilled birth attendant as “an accredited health professional – such as a midwife, doctor or nurse – who has been trained to proficiency in the skills needed to manage normal (uncomplicated) pregnancies, childbirth and the immediate postnatal period, and in the identification, management and referral of complications in women and newborns”(WHO, 2004). Several studies have shown evidence of an association between utilization of skilled attendants during childbirth and reduction in maternal deaths (Hogberg, Wall, & Brostrom, 1986; Fauveau, Stewart, Khan, & Chakraborty, 1991; De Brouwere, Tonglet, & Van Lerberghe, 1998; Hussein et al., 2004). Although there has been some improvement in the utilization of skilled birth attendants in Ghana and other countries in sub-Saharan Africa, recent maternal mortality estimates indicate that there are huge disparities in regional, country-level and within-country estimates (Boerma, Bryce, Kinfu, Axelson, & Victora, 2008; Bhutta et al., 2010; Hogan et al., 2010; Lozano et al., 2011).

In Ghana, the Minister of Health declared in 2008 that the country’s maternal mortality should be treated as a national emergency (MoH, 2008). Different strategies have been tried to reduce the high rate of maternal mortality by putting policies in place to ensure adequate antenatal care and improve the utilization of skilled birth attendants during childbirth. The most current and notable of these strategies is the introduction, in 2005, of free maternal health care for all pregnant women attending antenatal care or seeking to deliver in public health facilities (Witter, Adjei, Armar-Klemesu, & Graham, 2009). These policies have, to certain extent, improved maternal health in general, but the maternal mortality ratio in Ghana remains as high as 385 per 100,000 live births (WHO, 2013). The proportion of births attended by skilled birth attendants was 57% in 2008 (GSS, 2009), much below the targets set by the international community to have at least 80% of births attended by skilled birth attendants in 2005, 85% in 2010 and 90% in 2015.

Research has indicated poor availability of services as one factor in non-use of skilled attendants during childbirth (Koblinsky et al., 2006), but even in areas where these services are available certain groups of women, including the poor, those with low education and rural backgrounds, fail to access these services (Saxena, Vangani, Mavalankar, & Thomsen, 2013; Tsegay et al., 2013). Reasons for this include high direct and indirect cost of services, lack of transport or distance to health facilities, lack of knowledge about services provided, previous unpleasant experiences, and cultural issues (Koblinsky et al., 2006; Hounton et al., 2008). To the best of our knowledge, no study has analyzed the geographical variability in the non-utilization of skilled birth attendants in Ghana. This current study is therefore aimed at analyzing the risk of not having a skilled birth attendant during childbirth in women of different socioeconomic backgrounds in Ghana using spatial techniques.

## 2. Methods

### 2.1 Study Setting, Survey Data, and Ethical Approval

The study was conducted in Ghana, located at the coast of West Africa, south of the Sahara. Ghana has 10 administrative regions and a population of 24,658,823 (based on the 2010 census population estimate) in a total land area of 238,535 km^2^. Data for this study of skilled birth attendant utilization were drawn from the 2008 Ghana Demographic and Health Survey (GDHS) carried out by the Ghana Statistical Service and the Ghana Health Service. The study was designed to be representative of the 10 administrative regions (Table 1). As part of the survey, 4,916 women age 15–49 were interviewed (response rate 96%), out of which 2,144 had at least one birth experience and therefore formed our study sample. Ethical approval for the study was obtained from the Ghana Health Service Ethics Review Committee.

**Table 1:** Distribution of sample births without skilled birth attendant, by administrative region in Ghana, 2008 (N = 2108)

Administrative Region	Number and Percentage						

	Total Births	Without Skilled Birth Attendant		To Mother with Low Education		To Mother with Low Income	
Ashanti	318 (100%)	79	(24.8%)	291	(91.5%)	176	(55.3%)
Brong Ahafo	207 (100%)	75	(36.2%)	195	(94.2%)	156	(75.4%)
Central	157 (100%)	65	(41.4%)	143	(91.1%)	104	(66.2%)
Eastern	187 (100%)	69	(36.9%)	175	(93.6%)	128	(68.4%)
Greater Accra	204 (100%)	32	(15.7%)	151	(74.0%)	29	(14.2%)
Northern	296 (100%)	203	(68.6%)	282	(95.3%)	263	(88.9%)
Upper East	179 (100%)	97	(54.2%)	165	(92.3%)	154	(86.0%)
Upper West	210 (100%)	106	(50.5%)	199	(94.8%)	187	(89.0%)
Volta	175 (100%)	77	(44.0%)	158	(90.3%)	141	(80.6%)
Western	175 (100%)	72	(41.1%)	161	(93.1%)	114	(65.1%)
**Total**	**2108**	**875**		**1920**		**1452**	

**Figure 1:**
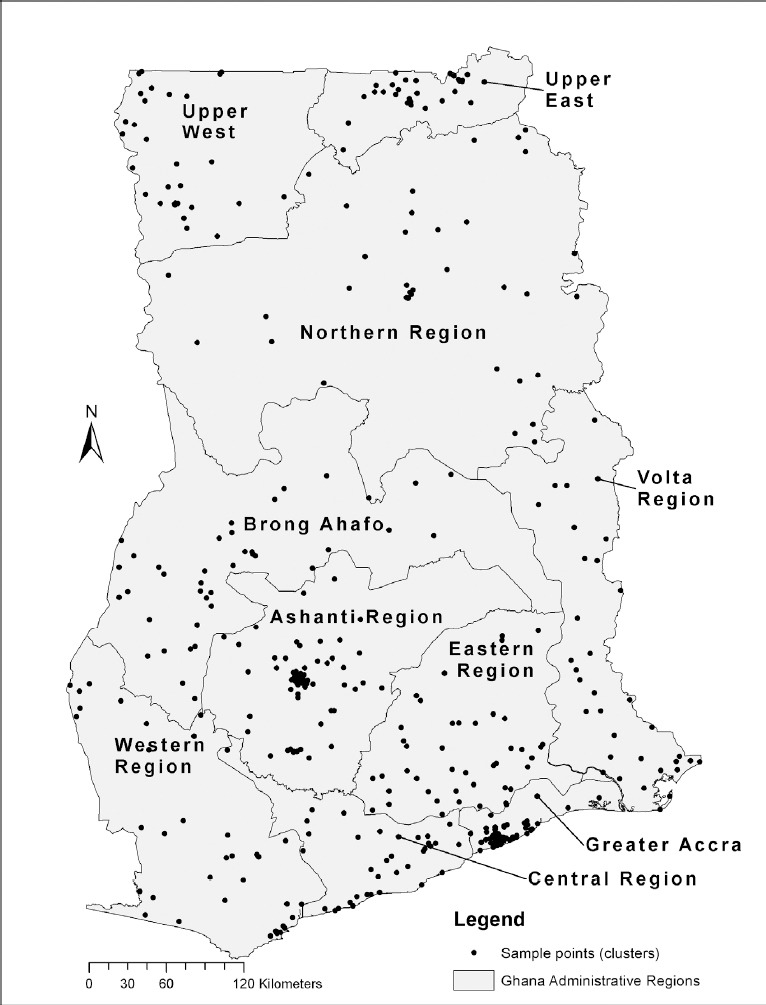
Study sample points (N = 404 clusters) by administrative region in Ghana

The survey collected geographic data at all sample points (called “clusters”). These geographic coordinates were later linked to the Demographic and Health Survey (DHS) dataset in our study. Women with at least one birth experience were found at 411 geographic clusters. Seven clusters with a total of 36 study respondents were excluded from the analysis because they had missing coordinates, bringing the final sample of women included in the study to 2,108 respondents spread across 404 clusters (Figure 1).

### 2.2 Study variables

Nine variables were used in the analysis: one outcome variable, two exposure variables, four confounding variables, and two geographic variables. The outcome variable was *lacking skilled attendant at birth*. This variable was generated from response to the question that assessed “the type of person that assisted in the delivery of the child.” The responses were dichotomized as *utilizing skilled attendant at birth* (Doctor, Nurse and Midwife) and *lacking skilled attendant at birth*. If a woman had more than one child, utilization of skilled birth attendant was assessed based on her latest birth experience during the study period.

The exposure variables included low education and low income. Low education was defined as having up to basic education. This included women who reported having no formal education and those with some formal education up to primary, middle school or lower secondary school (less than 12 years). The reference category for the education exposure variable includes women with 12 or more years of formal education. For the income exposure variable, income level was calculated based on the yearly earnings of the respondents. This variable was originally categorized into quintiles (poorest, poor, middle, rich, richest) according to the Ghana Demographic and Health Survey. Preliminary analysis of these income groups showed that women in 4^th^ and 5^th^ quintiles had similar and favourable health outcomes compared to those in the lower quintiles. The responses were therefore dichotomized as low income (poorest, poorer, middle) and high income (richer and richest) for this analysis. Four confounding variables: age (<25, 25–34, and 35+), marital status (single [never married, separated, divorced or widowed] and married [including cohabiting]), residence (rural/urban), and parity (nulliparous, para 1–3, para 4+), were included in a multivariate logistic regression analysis to adjust for potential confounding effects. This was to test for the independent association between low education, low income, and non-use of skilled birth attendants.

Finally, to conduct the spatial analyses, the longitude and latitude of each cluster location were required. These data were obtained from the database of Measure DHS and converted to the Ghana Metre Grid projected coordinate system using ArcGIS 10.1 software.

### 2.3 Analytical Methods

To investigate geographical variability in the association between non-utilization of skilled birth attendants and socioeconomic background of Ghanaian women, we used both “global” and “local” spatial statistics (Fotheringham, Brunsdon, & Charlton, 2002; Lloyd, 2006). Global here does not mean “worldwide.” Instead, it refers to measures summarizing the entire dataset. “Global” statistics summarize data for an entire study area to yield a single aggregate measure that sometimes provides misleading interpretations of local relationships. Global statistics are often aspatial, normally based on the assumption that the associations under investigation do not vary in space, and in many cases mask spatial variations. Local statistics, on the other hand, summarize data for individual places within the entire study area and therefore yield multiple statistics, one for each cluster in the sample (Fotheringham et al., 2002; Lloyd, 2006).

This study employed global and local or geographically weighted odds ratios to examine the spatially varying relationships between low socioeconomic status (low education and low income) and non-utilization of skilled birth attendants. The odds ratio is widely used in health research to explore the relationships between exposure and outcome. The formula for the odds ratios is:

OR = (a/b)/(c/d)=ad/bc

where a/b is the odds of being a case among the exposed and c/d represent the odds of being a case among the non-exposed.(Aschengrau & Seage, 2008)

Geographically weighted odds ratios were calculated for the education exposure variable for each cluster based on the following formula where *a_i_*, *b_i_*, *c_i_*, and *d_i_* are weighted totals for the ith cluster.

GWOR_i_ = *a_i_ d_i_/b_i_ c_i_*

where *a_i_* = ∑*_j_A_j_w_ij_ b_i_* = ∑*_j_B_j_w_ij_ c_i_* = ∑*_j_C_j_w_ij_ d_i_* = ∑*_j_D_j_w_ij_* and

A_j_ = number of women with low education who had no skilled birth attendant

B_j_ = number of women with high education who had no skilled birth attendant

C_j_ = number of women with low education who had a skilled birth attendant

D_j_ = number of women high education who had a skilled birth attendant

*w_ij_* = exp[-0.5(*d_ij_^2^/h^2^*)] = the weight using a Gaussian function

*where d_ij_* = the distance from cluster centroid i to cluster centroid *j*

*h* = the bandwidth

A similar formula was used to calculate the geographically weighted odds ratios for the income exposure variable.

In the literature on spatial weights, the effect of distance is expressed in terms of bandwidth, the distance describing the zone or kernel of interest around a cluster of interest, and kernel function describing how influence varies with distance. Different bandwidths can be used and different functions are available for modeling the effects of distance. The results of an analysis will change if the kernel function and/or bandwidth are changed.

Specifying the bandwidth sets a level between the level of the individual health event and all health events in the country as a whole, providing insight into the scale at which processes affecting maternal health outcomes are operating. For this study, a fixed bandwidth of 60 km was chosen in relation to the size of administrative regions

Based on the commonly-used Gaussian formula for spatial weights, at distances from one cluster location to another cluster that are less than the bandwidth, the ratio of distance squared to bandwidth squared is less than 1 and the weight is large. At distances from one cluster to another that are greater than the bandwidth, the weight formula ratio is greater than 1 and the value of the weight drops off rapidly as distance increases. If the distance from one cluster centroid to another equals the bandwidth, the ratio is equal to -1 and the weight is equal to 1/e^(0.5)^.

Global and local odds ratios were calculated in a program using proc iml, the interactive matrix language procedure, in SAS Version 9.3. The program reported raw global and local odds ratios and the natural log of each odds ratio. We used the natural log of the GWORs to address the problem of skewness. The sample OR is limited at the lower end since it cannot be negative, but widely spread at the upper end. The sample OR therefore has a skew distribution, a problem that is avoided when the log of the odds ratio is used (Bland & Altman, 2000). A multivariate logistic regression model was constructed in the aspatial analysis (in SPSS version 20), to adjust for possible confounding by age, marital status, residence, parity, and mutually for education and income.

## 3. Results

The non-use of skilled birth attendants in the study sample varied by education and income strata (Table 2). In our sample, 41.5% of the women lacked skilled attendants when giving birth. While 92.5% of women with secondary or higher education accessed skilled care at birth, only 55.2% of women with less education had skilled birth attendants. Similarly, 88.3% of high-income women utilized skilled attendants whereas only 45.0% of low-income women did. The global odds ratios (Table 3) show that having no or only basic level education was strongly associated with lack of skilled birth attendants (OR _crude_ (95% CI) = 8.7 (5.0-15.3); OR _adjusted_ (95% CI) = 3.8 (2.1 – 7.0), log OR 2.77), as was having low income (OR _crude_ (95% CI) = 8.2 (6.3 - 10.7); OR _adjusted_ (95% CI) = 3.3 (2.5 - 4.5), log OR 2.28).

**Table 2:** The distribution of non-utilization skilled birth attendant among Ghanaian women (N= 2108) stratified by age, marital status, residence, parity, education, and income

Variables	Lacked skilled attendant: No 1233 (58.5%)	Lacked skilled attendant: Yes 875 (41.5%)	Total, n (%)
**Age**			
< 25	288 (56.9)	218 (43.1)	506 (100)
25-34	599 (61.8)	371 (38.2)	970 (100)
35 +	346 (54.7)	286 (45.5)	2108 (100)
**Marital status**			
Single	153 (64.3)	85 (35.7)	238 (100)
Married	1080 (57.8)	790 (42.2)	1870 (100)
**Residence**			
Rural	586 (43.3)	767 (56.7)	1353 (100)
Urban	647 (85.7)	108 (14.3)	755 (100)
**Parity**			
Nulliparous	322 (71.9)	126 (28.1)	448 (100)
Para 1 – 3	650 (60.5)	424 (39.5)	1074 (100)
Para ≥ 4	261 (44.5)	325 (55.5)	586 (100)
**Educational attainment**			
Low (basic level or no education)	1059 (55.2)	861 (44.8)	1920 (100)
High (secondary +)	172 (92.5)	14 (7.5)	186 (100)
Total	1231	875	2106
Missing	2		
**Income Level**			
Low-income	654 (45.0)	798 (55.0)	1452 (100)
High-income	579 (88.3)	77 (11.7)	656 (100)
Total	1233	875	2108

**Table 3:** Global odds ratios for non-use of skilled birth attendance according to women’s age, marital status, residence, parity, education, and income level

Variable	Crude odds ratio (95% CI)	Adjusted odds ratio (95% CI)*	Log OR§(Range)
**Age**			
< 25	0.9 (0.7 – 1.1)	1.8 (1.2 – 2.7)	
25-34	0.8 (0.6 – 1.0)	1.2 (0.9 – 1.6)	
35 +	1 (reference)	1 (reference)	
**Marital status**			
Single	0.8 (0.6 – 1.0)	1.0 (0.7 – 1.4)	
Married	1 (reference)	1 (reference)	
**Residence**			
Rural	7.3 (5.7 – 9.2)	3.8 (2.9 – 4.9)	
Urban	1 (reference)	1 (reference)	
**Parity**			
Nulliparous	1 (reference)	1 (reference)	
Para 1 – 3	1.6 (1.3 – 2.0)	1.9 (1.4 – 2.7)	
Para ≥ 4	3.0 (2.3 – 3.9)	3.1 (2.0 – 4.8)	
**Education**			
Low	8.7 (5.0 – 15.3)	3.8 (2.1 – 7.0)	2.77 (0.75 – 9.26)
High	1 (reference)	1 (reference)	0 (reference)
**Income**			
Low	8.2 (6.3 – 10.7)	3.3 (2.5 – 4.5)	2.28 (1.11 – 6.34)
High	1 (reference)	1 (reference)	0 (reference)

* Adjusted mutually for one another in a multivariate logistic regression model in SPSS Version 20

§ Calculated in a program using proc iml, in SAS Version 9.3 (only for education and income)

**Figure 2:**
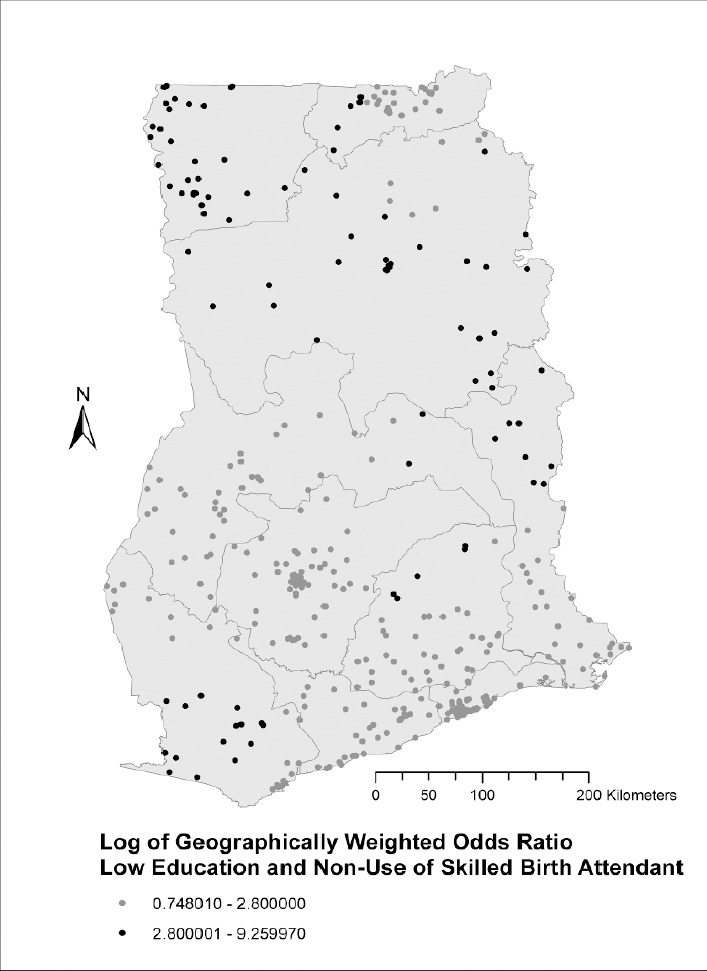
Spatial variation in the odds of not using a skilled attendant during delivery by women with no or up to basic level education in Ghana

The geographically weighted odds ratios reveal spatial variation in the association between non-use of skilled birth attendants and low education (Log of GWOR ranged from 0.75 to 9.26) or low income (Log of GWOR ranged from 1.11 to 6.34). An interesting pattern was observed for education. Women with no or only basic level education in Upper West, a large part of Northern region, the northern part of Volta region and the southern part of Western region had a higher odds of not using skilled birth attendants during delivery (Figure 2). All of the log GWOR values were greater than 0 so less education was associated with less use of skilled birth attendants everywhere, but the magnitude of that effect was spatially variable.

**Figure 3:**
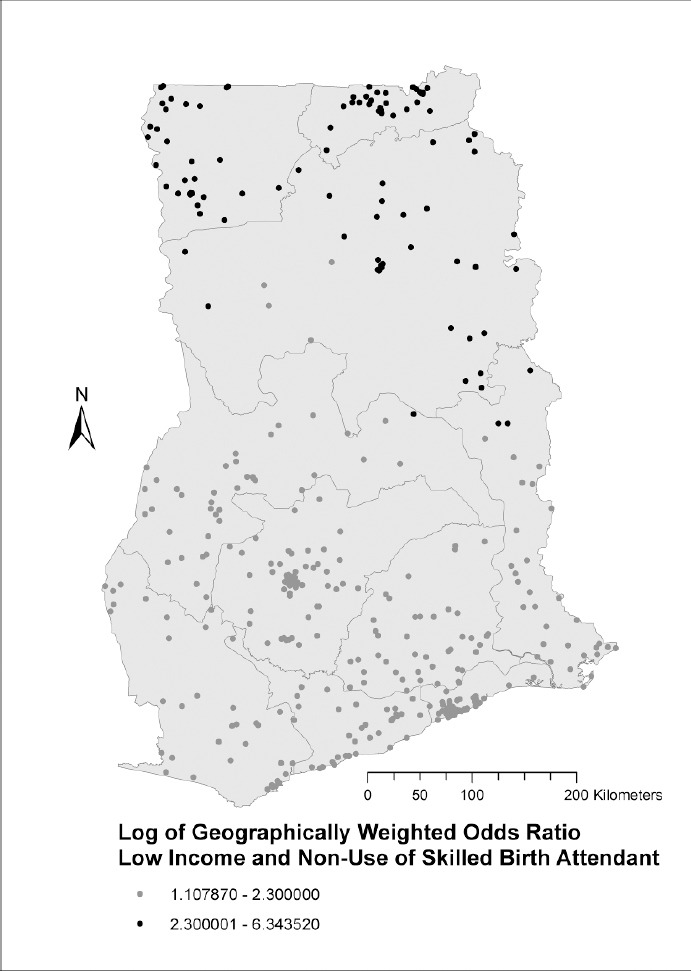
Spatial variation in the odds of not utilizing a skilled birth attendant by low-income women in Ghana

A different, north-south pattern was observed in the GWORs for income, with low-income women from the three northern regions (Upper West, Upper East and Northern region) having much higher odds of not using skilled birth attendants than women in other regions (Figure 3). Again, all of the log GWOR values were greater than 0 so low income was associated with less use of skilled birth attendants everywhere, but the size of that effect was spatially variable.

## 4. Discussion

The strength of associations between delivering without a skilled birth attendant and socioeconomic status (low education and low income) varies geographically in Ghana. In the Upper West region, some parts of Northern region, upper Volta and the southern part of Western region, less educated women have the highest odds of not utilizing skilled health professionals during delivery. In the northern sector of Ghana (Northern region, Upper East and Upper West regions), low-income women are at higher odds of not receiving skilled care during delivery compared to low-income women in other geographic areas in the country. Studies conducted in Kenya (Van Malderen et al., 2013) and Malawi (Makoka, 2009) also found some geographic variations in socioeconomic inequalities in utilization of skilled birth attendants.

The spatial distribution of the effects of low education on the non-use of skilled birth attendants varies slightly from that of low income. Aside from the Upper West region, the least educated women in the northern sector of Ghana including Upper East, greater parts of Northern region, and northern parts of Volta region are at the highest risk of delivering without the assistance of a skilled birth attendant. Less educated women in the southern parts of the western region also face a very high risk of delivering without professional assistance. Literacy in these regions, which are among the least developed geographic areas in Ghana, is generally lower than in the rest of the country (Senadza, 2012). A previous study has found that women in these geographic locations not only receive less education, but also lower quality education (Canagarajah & Xiao, 2001). Educational inequality is also higher in the poorer three northern regions. Females receive an average of 1.5 years of schooling there, while the remaining seven regions average 5.3 years (Senadza, 2012).

Apart from the distribution of socioeconomic infrastructure and resources that explain the non-use of skilled birth attendants in these settings, this observation could also be suggestive that high literacy levels in certain geographic areas may have a protective effect on illiterate or less educated women’s use of maternal health services, particularly skilled care at birth. Thus, in regions or localities with relatively low educational attainment, less educated women had a higher odds of not utilizing a skilled birth attendant during childbirth compared to similarly less educated women in the other regions or localities. This may be more visible in collective societies where social interactions are very strong and health seeking behavior of women, particularly, the decision to deliver with a skilled birth attendant, is not only dependent on the pregnant woman but also husbands, mother-in-laws, community heads, soothsayers, or traditional healers (Moyer et al., 2014). Thus, when women are educated to at least a senior high school level, their decisions and influence tend to affect the entire community, including less educated women. Thus, their use may encourage the use of maternal health services including SBA across all educational levels. Less educated women in the Upper East region, despite being vulnerable to the geographic and socioeconomic risk factors discussed above, still had a risk comparable to less educated women in the advantaged regions of southern Ghana. This may be because the Upper East region has been home to several health intervention researches or facilities, notably the Navrongo Health Research Centre, which has a special focus on maternal health services. These interventions have increased the awareness and use of maternal health services among less educated women in the region. A recent study conducted in Ghana found that the Upper East region has one of the shortest travel times to a birthing facility: 45 minutes or less, compared to 45 to 90 minutes in the Upper West, 180 to 360 minutes in the Northern Region, and more than 360 minutes in northern parts of Volta region (Masters et al., 2013).

The use of skilled birth attendants by low-income women in Ghana is dependent on the socioeconomic inequalities and varies considerably between southern and northern Ghana (Aryeetey, Owusu, & Mensah, 2009; Annim, Mariwah, & Sebu, 2012). Poverty in Ghana is concentrated in specific geographic areas. The three northern regions of the country, Upper West, Upper East and Northern regions are Ghana’s most deprived areas with regard to socioeconomic resources since they are far from the coast and seaports, and their climate is not suitable for growing cash crops (cocoa, coffee and rubber) (Owusu, 2005). They have also received less colonial and post-colonial investments. On the other hand, southern Ghana, which is enriched with exploitable and exportable resources, received many colonial and post-colonial investments. Southern Ghana has a climate suited for the cultivation of cocoa, coffee and rubber, the vegetation is suitable for timber exploitation and there are mining sites that are close to the coast and seaports. These geographic advantages allow southern Ghana to continue to attract investments (Owusu, 2005). This may be explained by Myrdal’s theory of cumulative causation (Myrdal, 1957). It recognizes that once a region takes the lead in socioeconomic development due to its comparative advantage (resulting from location and infrastructure), new economic activities and growth tend to be concentrated there, rather than in localities with fewer resources. In Myrdal’s view, government intervention is needed to address inequalities between advantaged and disadvantaged regions.

The government of Ghana has long recognized the socioeconomic inequality between northern and southern Ghana, but no policy has addressed this discrepancy effectively. Moreover, the health effects of these north-south spatial variations in Ghana have not been previously researched and there is little or no awareness of detrimental health consequences. The continuing state and private investments in regions with exportable products, and the provision of infrastructural support to those regions continue to promote inequalities in health outcomes among regions in Ghana (Aryeetey et al., 2009).

### 4.1 Methodological Consideration

The main strength of our study is its ability to use spatial techniques to study geographical inequalities in the use of skilled birth attendants in Ghana. Thus, it will contribute to scientific literature, public health practice and policy. However, using this technique limits the number of variables that can be included in the model, thereby limiting the possibility of adjusting for several possible confounders. We tried to overcome this weakness by first testing the independent association between our exposure variables (education and income) and non-use of skilled birth attendance in a multivariate logistic regression model. This made it possible for us to adjust for the effect of age, marital status, residence, and parity on such association. The study used self-reported DHS data that is prone to recall bias. Recall bias in reporting non-use of skilled birth attendants was reduced by analyzing only women’s latest birth experiences. Also, classification of income based on reported data is susceptible to biases. Women who work in the formal sectors may be more likely to report their income accurately than those in the informal sectors. Since women with low education are more likely to have informal occupation, their estimation of income may be less accurate. However, this may have no overall effect on our analysis since low-income women in our sample are fairly distributed across the 10 administrative regions (Table 1).

## 5. Conclusion

There is a spatially variable socioeconomic inequality in the use of skilled birth attendants in Ghana. Less educated women in the Upper West region, some part of Northern region, upper Volta and the southern part of Western region are the most at risk. Similarly, low-income women in the northern sector of Ghana are at the highest risk of not using skilled birth attendants during childbirth. Effective governmental and non-governmental interventions are needed to address such inequalities between the advantaged and the disadvantaged regions.
